# Stratification of Stage III colon cancer may identify a patient group not requiring adjuvant chemotherapy

**DOI:** 10.1007/s00432-020-03381-w

**Published:** 2020-09-13

**Authors:** Yasir G. Malik, Lars Gustav Lyckander, Jonas C. Lindstrøm, Olof Vinge-Holmquist, Ariba E. Sheikh, Johannes K. Schultz, Dejan Ignjatovic

**Affiliations:** 1 Department of Digestive Surgery, Akershus University Hospital, Sykehusveien 25, 1478 Lørenskog, Norway; 2 Institute of Clinical Medicine, Faculty of Medicine, University of Oslo, Oslo, Norway; 3 Department of Pathology, Akershus University Hospital, Lørenskog, Norway; 4 Health Services Research Unit, Akershus University Hospital, Lørenskog, Norway

**Keywords:** Stage III colon cancer, Adjuvant chemotherapy, Vascular invasion, Lymph node ratio, Complete mesocolic excision

## Abstract

**Purpose:**

Adjuvant chemotherapy for colon cancer with lymph node involvement (Stage III) has been the standard of care since the 1990s. Meanwhile, considerable evolvement of surgery combined with dedicated histopathological examinations may have led to stage migration. Furthermore, prognostic factors other than lymph node involvement have proven to affect overall survival. Thus, adjuvant chemotherapy in Stage III colon cancer should be reconsidered. The objective was to compare recurrence rates and survival in stage III colon cancer patients treated with or without adjuvant chemotherapy. Further, to assess the impact of extensive mesenterectomy, lymph node stage and vascular invasion on outcome.

**Methods:**

Consecutive patients operated for Stage III colon carcinoma between 31 December 2005 and 31 December 2015 were identified in the pathological code register by matching colon (T67) and either adenocarcinoma (M81403) or mucinous adenocarcinoma (M84803), with lymph node (T08) and metastasis of adenocarcinoma (M81406 or M84806). Medical records of all identified patients were reviewed.

**Results:**

Of 216 identified patients, 69 received no postoperative adjuvant chemotherapy (group NC), 69 insufficient adjuvant chemotherapy (FLV or < minimum recommended 6 cycles FLOX, group IC), and 78 sufficient adjuvant chemotherapy (≥ 6 cycles FLOX, group SC). When adjusted for age and comorbidity, 5-year overall survival did not differ statistically significant between groups (76% vs. 83% vs. 85%, respectively). Vascular invasion and a high lymph node ratio significantly reduced overall survival.

**Conclusion:**

The findings imply that subgroups of Stage III colon cancer patients have good prognosis also without adjuvant chemotherapy. For definite conclusions about necessity of adjuvant chemotherapy, prospective trials are needed.

## Introduction

Colon cancer is among the most common cancers worldwide (GBD 2017 Colorectal Cancer Collaborators 2019). In the past 60 years, the incidence of colon cancer in Norway has increased substantially, more prominently than in other Scandinavian countries. Since the 1970s, the 5-year relative survival has steadily improved, from under 30% in the 1960s to over 60% at present. The 5-year relative survival is above 90% for localized tumor (Stage I–II), and about 80% for locally advanced (Stage III) cancer, but only 10–20% in cases with metastatic disease. The current Norwegian treatment algorithm for Stage III disease consists of surgery (central vessel ligation encouraged, but the extent of the mesenterectomy remains undefined) with addition of adjuvant chemotherapy if the patient is under 75 years of age. Patients between 70 and 75 years are usually offered monotherapy with either capecitabine (Xeloda) or 5-fluorouracil (FLV) whereas combination therapy is recommended for patients under the age of 70 years [N1: XELOX (6 cycles of capecitabine + oxaliplatin], N2: XELOX/FOLFOX/FLOX (12 cycles of 5-fluorouracil + oxaliplatin) (Helsedirektoratet 2019). These recommendations are mainly based on older studies (Laurie et al. 1989; Moertel et al. 1990). The treatment algorithm is adjusted in case of comorbidity or poor tolerance, but is otherwise not personalized.

Today, we are complicit witnesses of several attempts to personalize treatment for colon cancer, with the aim to improve outcomes. One such example is immunotherapy for microsatellite-instable (MSI) tumors (Weger et al. 2012). It is estimated that adjuvant treatment in Stage III disease improves 5-year overall survival (OS) by 7–8% (Gill et al. 2004). However, many other risk factors for recurrence apart from N stage have been identified [i.e., vascular invasion (Leijssen et al. 2019), T stage (Li et al. 2018), perineural invasion (Yang et al. 2015), genetic factors (Antelo et al. 2012)] and it is quite likely that the effect of adjuvant therapy varies considerably within the Stage III group. Furthermore, surgery has evolved towards more radical methods since the introduction of adjuvant chemotherapy and some Stage III patients might, therefore, be overtreated when following current guidelines for adjuvant chemotherapy.

The aim of this study was to compare OS and recurrence rates after surgery for Stage III colon cancer in patients who did/did not receive adjuvant chemotherapy. Furthermore, the impact of the extent of mesenterectomy as well as lymph node stage and vascular invasion (VI) was assessed.

## Materials and methods

In this single-center quality control cohort study, all patients operated for colon cancer with lymph node involvement between 31 December 2005 and 31 December 2015 at Akershus University hospital were eligible. Exclusion criteria were distant metastasis at the time of diagnosis, tumor perforation, death due to postoperative complications, non-radical surgery (R1, R2), relocation to an address outside the Akershus University hospital recruitment area after surgery and insufficient CT staging (emergency surgery).

All patients treated for Stage III colon cancer during the study period were identified from the pathology laboratory information system (DocuLive Pathology, Cerner) using a search module (Pat Stat) to identify SNOMED codes for topography (T-code T67 for colon) and morphology (M-code M81403 for adenocarcinoma or M 84803 for mucinous adenocarcinoma). All of these patients who at the same time period were registered with lymph node (T08) combined with metastasis of either adenocarcinoma (M81406) or mucinous adenocarcinoma (M84806) were included in the trial. The lists of patients registered with these codes were provided by the pathologist and handled by the Data capture group at Akershus University Hospital.

Clinical data were collected by review of patient files (YM, OV-H and AS) using the electronic medical record system DIPS (Copyright 1995–2016 DIPS ASA version 7.395). The baseline variables included demographic data, ASA (American Society of Anesthesiologists) classification (ASA 2014) and Charlson comorbidity index (CCI) (Charlson et al. 1987). Treatment-related variables included data concerning the surgical intervention and adjuvant chemotherapy, including types and number of received chemotherapy cycles, which in turn laid the foundation for the study population to be divided into three groups:

Group 1: no chemotherapy (NC), Group 2: insufficient chemotherapy (IC, i.e., less than minimum recommended in Norway—either FLV (monotherapy) or less than six cycles of FLOX), Group 3: sufficient chemotherapy [SC; a minimum of six cycles of FLOX as recommended (Iveson et al. 2019)]. The extent of the lymph node dissection (D2 or D3) was registered. For left-sided colectomy and sigmoid resection, D3 was defined as central IMA (inferior mesenteric artery) ligation (proximal to the left colic artery). A right-sided colectomy was defined as D3 only if all lymphatic tissue anterior and posterior to the superior mesenteric vessels was removed, as for patients included in the clinical trial “Safe Radical D3 Right Hemi colectomy for Cancer through Preoperative Biphasic Multi-Detector Computed Tomography (MDCT) Angiography” (ClinicalTrials.gov identifier: NCT01351714) (Gaupset et al. 2018; Nesgaar et al. 2019), which enrolled patients since 2011 at Akershus University Hospital.

Data about tumor biology included tumor size, differentiation, morphology, total number of lymph nodes removed, number of lymph nodes with metastasis, T stage (TNM classification, 8th edition) (Brierley Wittekind 2017) and vascular invasion (VI). VI was not regularly reported during the study period and did not differentiate between extramural vascular invasion (EMVI) and intramural vascular invasion (IMVI). A single experienced GI-pathologist (LGL) reanalyzed the original slides of patients with missing data on vascular invasion, according to the latest guidelines of The Royal College of Pathologists (RCPath 2020).

The outcome measures included type of recurrence based on first CT scan showing recurrence, time to last CT scan during the follow-up and time to death.

Ethical approval for this study was applied for at the Regional Ethics Committee (REC) South East Norway. The reply stated that ethical approval was not required as this is a local retrospective quality control study (Reference 2016/1285 REC South East B). Approval was given by the data protection officer (nr: 16-128) at Akershus University Hospital.

### Statistical analysis

Demographic variables were analyzed using *t* tests and chi-square test. Time from surgery to recurrence or death was analyzed using Cox regression modeling. In time-to-recurrence analyses, deaths were censored. Adjustments for age and comorbidity (CCI) were done by adding them as covariates in the Cox model. The Cox models were also used to estimate 5-year survival, and adjusted 5-year survival estimates were presented for age = 62 and CCI = 2.5, which were about the mean values for these variables. All statistical analyses were done in R version 3.6 (R core team 2013).

## Results

**Fig. 1:**
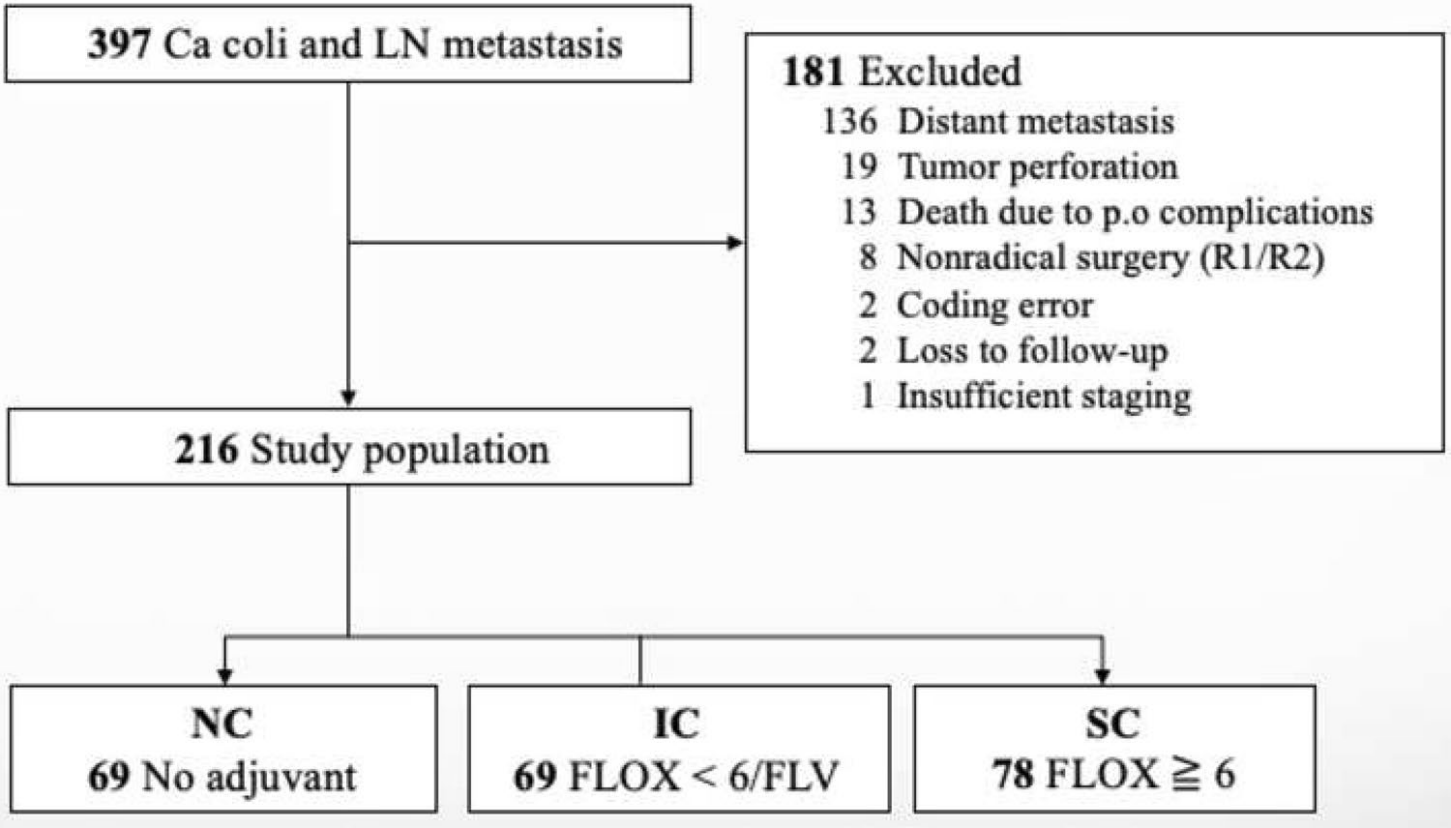
Study flow diagram with exclusions

A total of 397 patients operated for colon cancer with lymph node metastasis during the study period were identified. After the exclusion of 181 patients (various reasons, Fig. 1), 216 patients were eligible for inclusion, of these 69 patients (group NC) did not receive chemotherapy, 69 patients (group IC) received either FLV or < 6 cycles FLOX, and the remaining 78 patients (group SC) received at least 6 FLOX cycles, Fig. 1.

Descriptive statistics for the baseline characteristics of the three groups are presented in Table 1. Statistically significant differences between the groups were found in age (*p* < 0.001 between all three groups) and CCI scores (*p* < 0.002 between all three groups). In the SC group, 91% of patients had no comorbidity at all compared to 72% in the IC group and 42% in the NC group.

**Table 1:** Baseline characteristics

	All patients (*n* = 216)	Group NC^a^ (*n* = 69)	Group IC^b^ (*n* = 69)	Group SC^c^ (*n* = 78)
Sex				
Male	95 (44%)	38 (55%)	27 (39%)	30 (39%)
Female	121 (56%)	31 (45%)	42 (61%)	48 (61%)
Age, mean (SD) (years)	67.5 (11.4)	77.8 (7.2)	67.2 (7.2)	58.8 (9.9)
BMI, mean (SD) (kg/m^2^)	25.3 (4.5)	24.6 (3.7)	25.7 (5.3)	25.4 (4.4)
Missing	23 (11%)	9 (13%)	6 (9%)	8 (10%)
Comorbidity				
None	150 (69%)	29 (42%)	50 (72%)	71 (91%)
Ischemic heart disease or heart failure	25 (12%)	17 (25%)	6 (9%)	2 (3%)
Chronic pulmonary disease	9 (4%)	6 (9%)	2 (3%)	1 (1%)
Cerebrovascular disease	12 (6%)	11 (16%)	0 (0%)	1 (1%)
Diabetes	21 (10%)	9 (13%)	9 (13%)	3 (4%)
Other comorbidity	17 (8%)	10 (14%)	7 (10%)	0 (0%)
ASA physical status score ^d^				
I	16 (7%)	1 (1%)	4 (6%)	11 (14%)
II	66 (31%)	17 (25%)	21 (30%)	28 (36%)
III	49 (23%)	25 (36%)	20 (29%)	4 (5%)
IV	4 (2%)	4 (6%)	0 (0%)	0 (0%)
Missing	81 (37%)	22 (31%)	24 (35%)	35 (45%)
CCI score, mean (SD)	2.5 (0.8)	2.9 (1.0)	2.4 (0.7)	2.1 (0.3)
Tumor localization				
Caecum, ascending colon	82 (38%)	27 (39%)	24 (35%)	31 (40%)
Transversum, flexures	51 (23%)	18 (26%)	19 (27%)	14 (18%)
Sigmoid, descending colon	83 (38%)	24 (35%)	26 (38%)	33 (42%)

*SD* standard deviation, *BMI* body-mass index, *ASA* American Society of Anesthesiologists, *CCI* Charlson comorbidity index

^a^Group NC = No adjuvant

^b^Group IC = FLOX < 6 or FLV

^c^Group SC = FLOX ≥ 6

^d^Anesthesiological comorbidity score; score range I to V; I indicates completely healthy, V indicates moribund

The results of surgery and outcomes are presented in Table 2. T stage, VI and D2/D3 dissection were equally distributed between the groups. Regarding tumor biology, 16% of the patients had low differentiated adenocarcinoma in the NC group, 23% in the IC group, and 10% in the SC group; these differences were not statistically significant and the groups were comparable for tumor size and morphology. Mean total lymph node harvest was comparable in all three groups (23.2 group NC; 25.4 group IC and 27.4 group SC). Significantly more lymph nodes were harvested with D3 dissection compared to D2 dissection (36.3 vs 21.9; *p* < 0.001). There were less patients with N2 status in group NC (26%) and IC (24%) compared to group SC (39%); these differences were not statistically significant. Lymph node ratios (metastatic lymph nodes/harvested lymph nodes) in the three groups were comparable. No significant difference in lymph node ratio (LNR) was found for patients with D2 dissection (0.18) compared to D3 dissection (0.12). There was a significant difference in recurrence rate between groups NC and SC (39% vs 17%; *p* = 0.004), and no significant difference between groups NC and IC (39% vs 29%) and IC and SC. Of 60 patients with recurrences, 16 were operated for their recurrence with curative intent (group NC: 4, group IC: 5, group SC: 7) and six of these did not have any further relapse (group NC: 1, group IC: 2, group SC: 3).

**Table 2:** Surgery and outcomes

	All patients (*n* = 216)	Group NC^a^ (*n* = 69)	Group IC^b^ (*n* = 69)	Group SC^c^ (*n* = 78)
Surgery				
D2 dissection	163 (75%)	53 (77%)	48 (70%)	62 (79%)
D3 dissection	53 (25%)	16 (23%)	21 (30%)	16 (21%)
Total LN, mean (SD)	25.4 (15.3)	23.2 (14)	25.4 (16.4)	27.4 (15.2)
Positive LN				
N1a	71 (33%)	28 (41%)	24 (35%)	19 (24%)
N1b	80 (37%)	23 (33%)	28 (41%)	29 (37%)
N2	65 (30%)	18 (26%)	17 (24%)	30 (39%)
LN ratio, mean (SD)	0.16 (0.16)	0.19 (0.20)	0.14 (0.12)	0.16 (0.14)
Vascular invasion	95 (44%)	29 (42%)	32 (46%)	34 (44%)
Missing	4 (2%)	3 (4%)	1 (1%)	0 (0%)
T-stage^d^				
T1	3 (1%)	0 (0%)	0 (0%)	3 (4%)
T2	16 (7%)	4 (6%)	5 (7%)	7 (9%)
T3	137 (63%)	47 (68%)	43 (62%)	47 (60%)
T4	47 (22%)	14 (20%)	17 (25%)	16 (21%)
T4b	12 (6%)	4 (6%)	4 (6%)	4 (5%)
Follow-up in months				
To last CT, mean (SD)	40.0 (25.2)	25.2 (18.7)	43.9 (25.4)	48.5 (24.6)
Missing	13 (6%)	8 (12%)	4 (6%)	1 (1%)
Clinical, mean (SD)	63.9 (39.2)	42.6 (30.2)	65.8 (39.4)	81.0 (37.4)
Recurrence^e^				
Total	60 (28%)	27 (39%)	20 (29%)	13 (17%)
Liver metastasis	17 (28%)	10 (37%)	3 (15%)	4 (31%)
Lung metastasis	6 (10%)	0 (0%)	4 (20%)	2 (15%)
Peritoneal Carsinomatosis	8 (13%)	4 (15%)	2 (10%)	2 (15%)
Local recurrence	2 (3%)	1 (4%)	1 (5%)	0 (0%)
Multiple metastasis	14 (23%)	7 (26%)	5 (25%)	2 (15%)
Other metastasis	13 (22%)	5 (19%)	5 (25%)	3 (23%)

*SD* standard deviation, *LN* Lymph node, *N1a* tumor cells in 1 regional lymph node, *N1b* tumor cells in 2 or 3 regional lymph nodes, *N2*: tumor cells in more than 3 regional lymph nodes, *T-stage* tumor stage

^a^Group NC = No adjuvant

^b^Group IC = FLOX < 6 or FLV

^c^Group SC = FLOX ≥ 6

^d^T-stage not defined for 1 patient in Group SC

^e^based on first CT scan showing recurrence

**Fig. 2:**
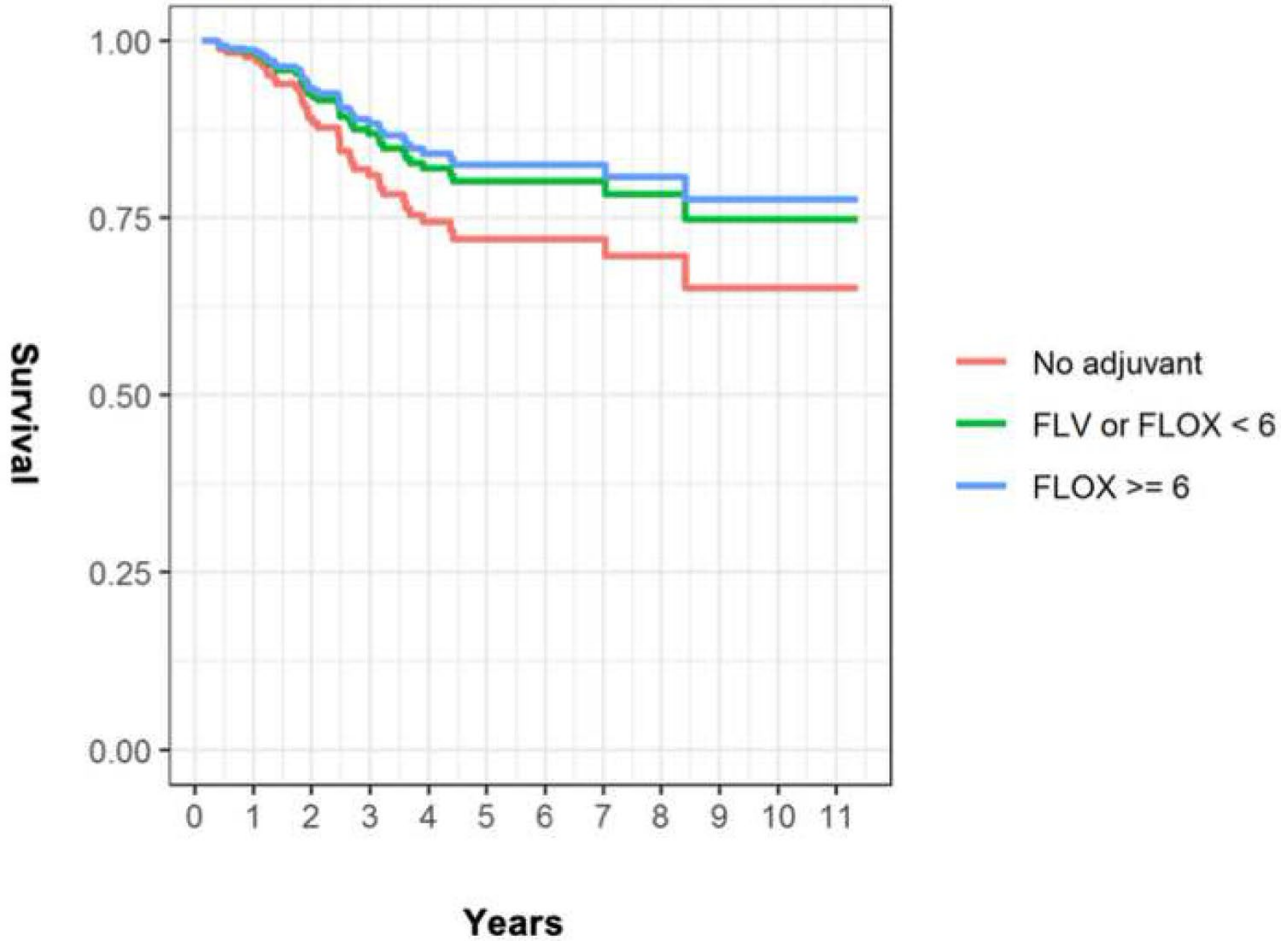
Estimated 5-year overall survival adjusted for age and CCI (age = 68 and CCI = 2.5) based on Cox regression

The crude 5-year OS (44% for group NC, 76% for group IC and 86% for group SC) differed significantly between groups SC and NC (HR: 3.09, CI: 1.40–6.80, *p* = 0.005) and not significantly between groups SC and IC (HR: 1.48, CI: 0.63–3.48, *p* = 0.37). After adjusting for age and CCI no significant differences were found in 5-year OS (group NC: 76%, group IC: 83%, group SC: 85%; between groups SC and NC: HR 1.70, CI: 0.53–5.42, *p* = 0.37; between groups SC and IC: HR 1.15, CI: 0.45–2.92, *p* = 0.78), Fig. 2.

**Fig. 3:**
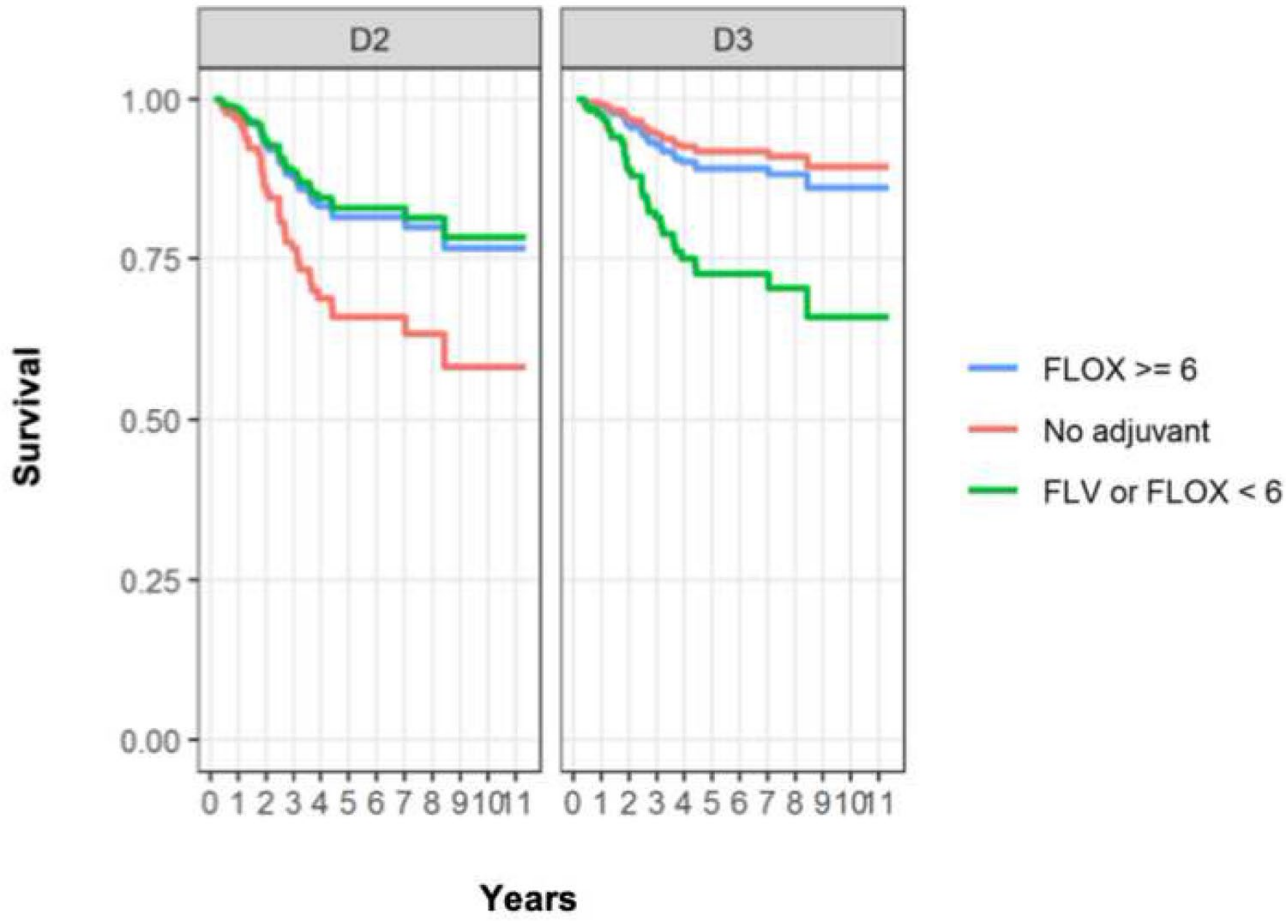
Estimated 5-year overall survival for D2 and D3 operated patients adjusted for age and CCI (age = 68 and CCI = 2.5) based on Cox regression

After correction for age and CCI, 5-year OS for patients operated with D2 or D3 dissection was 71% vs 93% in group NC (HR: 0.37, CI: 0.02–6.73, *p* = 0.50), 86% vs 77% in group IC (HR: 3.06, CI: 0.28–34.02, *p* = 0.36) and 85% vs 91% in group SC (HR: 0.56, CI: 0.07–4.41, *p* = 0.58), Fig. 3.

**Fig. 4:**
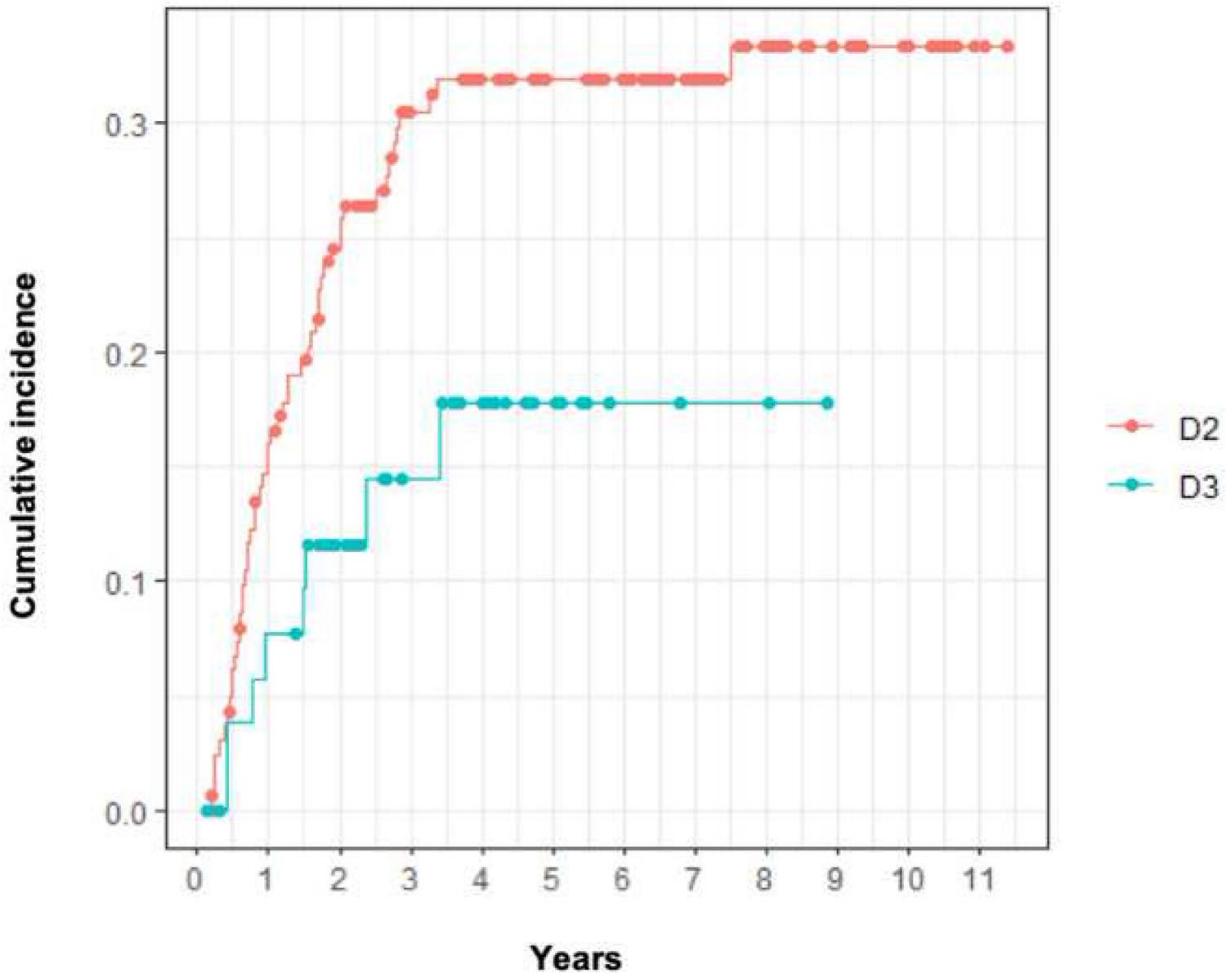
Time to recurrence in D2 and D3 operated patients

The observed difference in time to recurrence between D2 and D3 operated patients (HR: 0.47, CI: 0.22–0.99, *p* = 0.047) was statistically significant, Fig. 4.

**Fig. 5:**
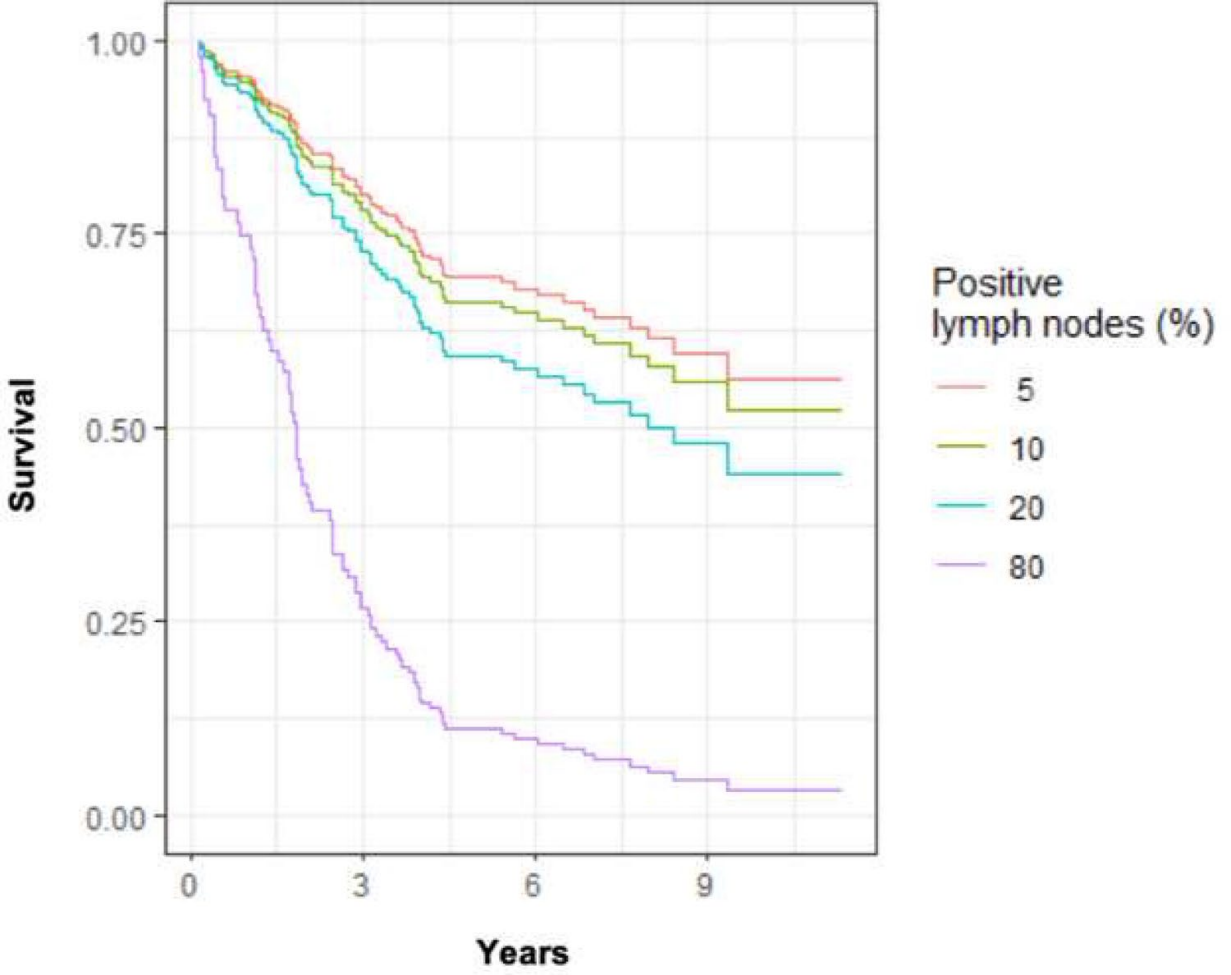
Estimated 5-year overall survival for different lymph node ratios based on Cox regression

The estimated 5-year overall survival depending on the LNR was 76% for LNR 0.05; 74% for LNR 0.1; 68% for LNR 0.2 and 20% for LNR 0.8, Fig. 5.

**Fig. 6:**
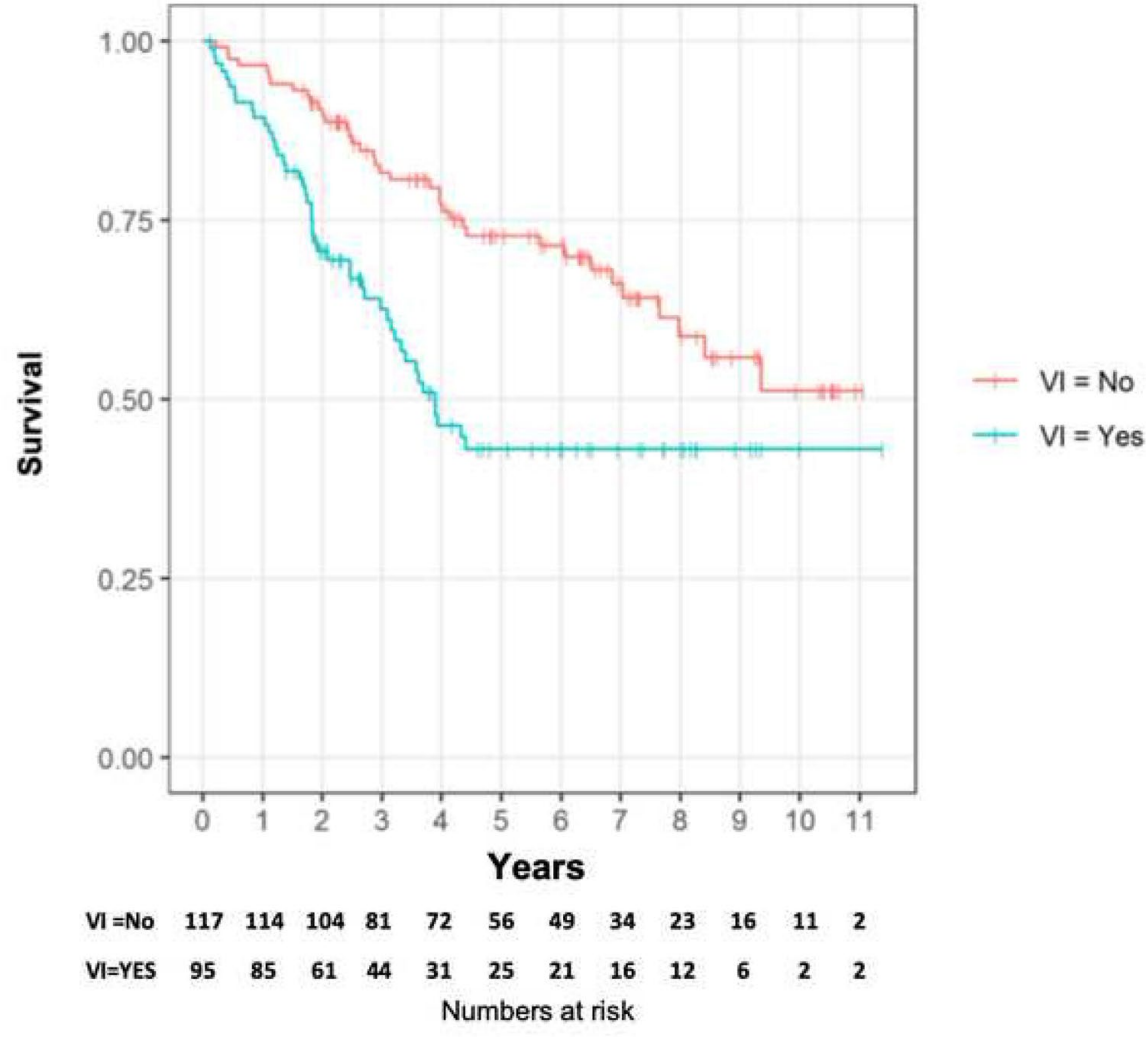
Kaplan–Meier curve showing 5-year overall survival for patients with or without vascular invasion

The difference in 5-year overall survival for patients with VI (52%) and without VI (81%) was significant (HR: 2.16, CI: 1.40–3.34, *p* = 0.001), Fig. 6.

**Fig. 7:**
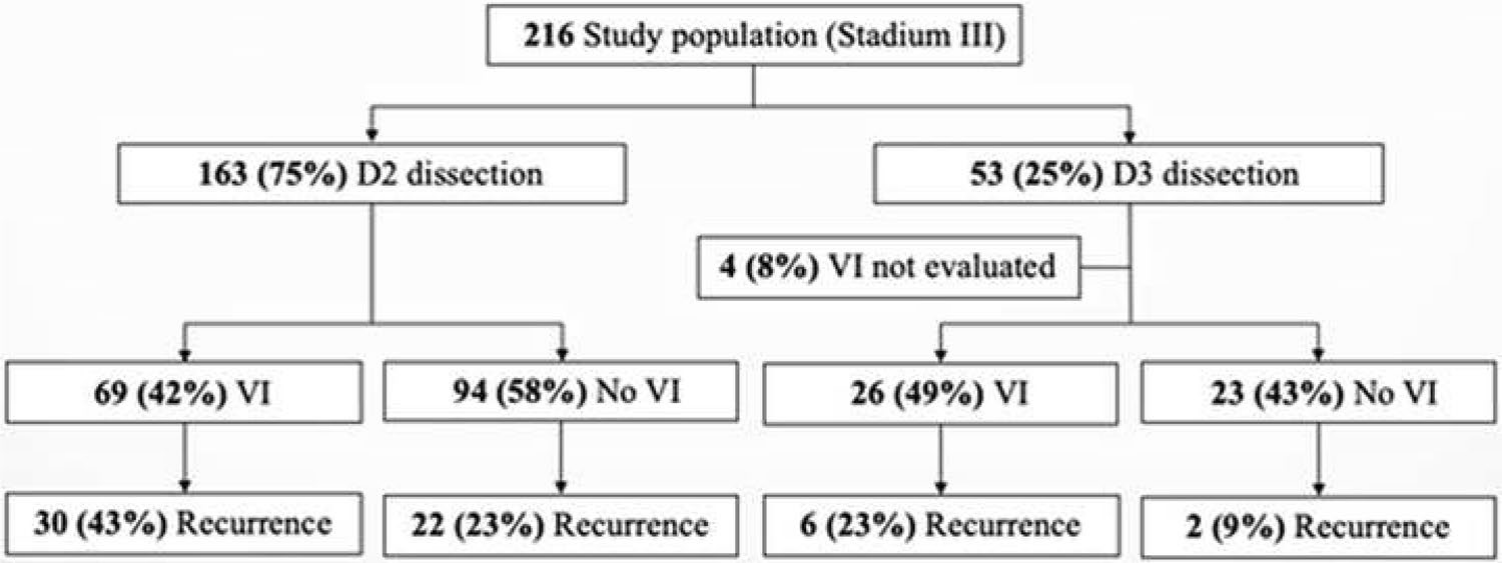
Flow diagram of correlation between D2/D3 dissection and vascular invasion

D3 dissection was performed on 7 patients (13%) between 2005 and 2010, and on 46 patients (87%) between 2011 and 2015. When broken down according to the extent of lymph node dissection (D2/D3) and VI (yes/no), the results presented in Fig. 7 demonstrate that the recurrence rate in patients with VI was 23% when operated with D3, and 43% when operated with D2. In VI negative patients, recurrences occurred in 9% of the patients after D3 operation and 23% after D2 operation. These differences were not statistically significant.

## Discussion

This single-center retrospective cohort study showed crude 5-year overall survival (OS) in patients operated for Stage III colon cancer was best after adjuvant chemotherapy. However, when adjusted for age and comorbidity, the differences between the groups were not statistically significant. Vascular invasion (VI) and lymph node ratio (LNR) had a significant impact on overall survival as independent prognostic factors. Time to recurrence was significantly longer after more extensive lymph node dissection (D3) compared to less extensive lymph node dissection (*p* = 0.047); however, the extent of lymph node dissection did not have a significant impact on 5-year OS.

In Norway, adjuvant chemotherapy is generally recommended after operation for Stage III colon cancer (Helsedirektoratet 2019). Comorbid and older patients (> 75 years) are usually not offered adjuvant chemotherapy, and a few patients even deny chemotherapy due to various reasons. This creates a significant selection bias in this cohort with effect on survival, which is clearly reflected in the results of this study. This explains why the large difference in crude overall survival is not statistically significant after the adjustment for age and CCI. One may argue that the observed difference in adjusted 5-year OS was not significant due to the small sample size. However, our findings are in line with a Scandinavian multicenter randomized trial, published in 2005, comparing surgery alone to surgery with adjuvant chemotherapy which showed no significant difference in OS (Glimelius et al. 2005).

The first recommendations for adjuvant therapy from the US National Cancer Institute (NCI) Consensus Conference (Adjuvant therapy for patients with colon and rectum cancer 1990) in 1990 were mainly based on two studies (Laurie et al. 1989; Moertel et al. 1990). Later, mostly the differences between different adjuvant regimes have been investigated. It is quite likely that the operative technique has become more radical since the RCTs conducted in the 1990s. The increased focus on lymph node harvest throughout the years has also resulted in a more intense search for lymph nodes within the specimen by the pathologist, possibly leading to stage migration and more extensive use of adjuvant chemotherapy. Furthermore, other risk factors than lymph node involvement have been shown to play a significant role for prognosis. In the light of this and our findings, one must question whether lymph node involvement alone should prompt adjuvant therapy in the future.

EMVI has been recognized to be a strong predictor for poor oncologic outcome in Stage II–III colon cancer patients, while IMVI is not. This association seems to be stronger than that for Lymph node involvement (Leijssen et al. 2019). In our study, a certain distinction between EMVI and IMVI was only used for the 110 patients for which histology was reanalyzed. However, none of these patients had IMVI. According to the pathology department, the vast majority of patients registered with VI in the original pathology report most likely had extramural invasion.

The quality of the surgical specimen and the lymphadenectomy are critical factors when outcomes are of concern; it is, however, rarely reported in papers on adjuvant treatment. Most of the studies showing significantly improved OS for Stage III colon cancer after adjuvant chemotherapy failed to show the same for Stage III rectal cancer (Laurie et al. 1989; Moertel et al. 1990). One reason may be the introduction of total mesorectal excision (TME) following the “holy plane” during the 90s, which leads to a decisive improvement of oncologic outcomes (Heald 1988). In fact, ESMO (European Society of Medical Oncology) does recommend not to base the decision for adjuvant chemotherapy of rectal cancer patients only on positive lymph nodes in the mesorectum (Glynne-Jones et al. 2017) and there is substantial variation in the use of this treatment in clinical practice (Bregni et al. 2020). Recently, “complete mesocolic excision” (CME) which follows the same principles as TME has been introduced for colon cancer, and several studies worldwide show a trend towards better survival (Hohenberger et al. 2009; Bertelsen et al. 2015).

As new knowledge on the spreading mechanisms of colon cancer is acquired, consciousness of the extent of mesenterectomy is in focus. It is noted in the literature that a minimum of 2–4% of all patients with colon cancer have centrally located lymph node metastases (Kim et al. 2004). Very few authors have a clear anatomical definition for these nodes (Spasojevic et al. 2013; Nesgaard et al. 2018). Today, the Norwegian national guidelines encourage that D3 (extended lymphadenectomy with central vessel ligation) should be performed, while D2 (limited lymphadenectomy with intermediate ligation of colonic vessels) represents the minimal requirement (Helsedirektoratet 2019). Several studies have shown that higher number of lymph nodes in the specimen are associated with better survival (Voyer et al. 2003; Swanson et al. 2003; Chen and Bilchik 2006; Kelder et al. 2009; Goldstein 2002). This entails more attention to the LNR in the future, which has a significant impact on survival as clearly illustrated in our study (Fig. 5), in accordance with previous studies (Berger et al. 2005; Rosenberg et al. 2008).

D3 dissection was a rarity in the first half of the study period but gradually became more common in the second half of the study period (13% vs 87%), mainly due to the introduction of a standardized approach with central vessel ligation for left-sided tumors and the start of the clinical trial “Safe Radical D3 Right Hemi-colectomy for Cancer through Preoperative Biphasic Multi-Detector Computed Tomography (MDCT) Angiography” (Gaupset et al. 2018; Nesgaar et al. 2019). In this trial, extensive lymphadenectomy is performed removing not only tissue located anterior but also posterior to the superior mesenteric vessels, this not being the case in D2 dissection. A recent study where conventional laparoscopic right hemi-colectomy was performed has shown that patients with an ileocolic artery (ICA) crossing posteriorly to the superior mesenteric vein (SMV) have worse disease-free survival (DFS) when compared to those with ICA crossing anteriorly (Ishiyama et al. 2020). It has recently also been demonstrated that lymph node distribution anterior or posterior to the superior mesenteric vessels highly depends on the crossing pattern of the ICA (Spasojevic et al. 2013; Nesgaard et al. 2018), which implies that inadequate lymphadenectomy (D2 dissection) may be the reason for worse outcome in patients with a posterior ICA crossing.

Our results show a tendency to better survival after D3 dissection compared to D2 dissection in the NC group, while there is not much difference in the two groups which received adjuvant chemotherapy. The observed recurrence rates in patients operated with D3 compared to D2 were also lower both for patients with or without VI (Fig. 7). Although not statistically significant, these differences might indicate better outcome for patients operated with D3 dissection.

Some studies have shown that the presence of circulating tumor DNA (ctDNA) after resection of Stage II and III colon cancer is associated with higher risk of recurrence (Tie et al. 2016, 2019). However, recurrences were also detected in patients where ctDNA was not found, underlining the importance of combining other prognostic factors (discussed in this paper) with ctDNA to help in adjuvant treatment decisions in the future.

This study has several weaknesses mostly owing to its retrospective design. One major drawback of the design is that the patient groups are not comparable with respect to age and comorbidity due to a well-established clinical practice for adjuvant chemotherapy based on the Norwegian national guidelines. There is also a slight possibility that patients were treated for recurrences elsewhere. However, as cancer treatment and emergency surgery for patients in Norway usually are done at the local hospital, treatment at other sites are normally reported to the local hospital. Patients (*n* = 2) who moved out of the catchment area of Akershus University Hospital were, therefore, excluded. Furthermore, recurrences appearing after the last registered CT scan may have occurred undetected. Nevertheless, this was a pilot study to evaluate the feasibility of omitting adjuvant chemotherapy in selected patients with Stage III colon cancer and a retrospective design was, therefore, the most appropriate.

As the sample size was small, no definite conclusions can be drawn from this study. One could argue that a multicenter design or a registry study would have gathered a larger sample size; however, the information about individual patients in the registries is not detailed enough for the purpose of this study. The main strength of the study is the inclusion of all consecutive Dukes C patients who fulfilled the inclusion criteria in a given time period increasing the external validity of the study.

## Conclusion

The retrospective design and limited sample size of this study precludes any conclusions about the necessity of chemotherapy. However, the findings imply that prognosis for subgroups of Stage III colon cancer patients is good also without chemotherapy, which in turn bears a significant risk of morbidity and even mortality (Andre et al. 2004). Prospective trials to evaluate the benefit of chemotherapy for these subgroups of Stage III colon cancer are needed.

## Data Availability

Available upon request.
